# Strategic exploration of the COVID-19 prevention campaign message: based on South Koreans’ perception type

**DOI:** 10.1186/s12889-022-13671-2

**Published:** 2022-06-28

**Authors:** Won Joo Choi, Jang Sun Hong

**Affiliations:** Department of Mass Communication, Konkuk University, Chungju-si, Republic of Korea

**Keywords:** COVID-19, Prevention Campaign, Public Message, Campaign Strategy, Q methodology

## Abstract

**Background:**

Many questions have been raised in the ongoing battle against COVID-19: How does the public perceive the COVID-19 prevention campaign as a member of the community?; What made the perception of the experts and the public on COVID-19 change from ‘simple’ to ‘serious’ epidemic?; What is the risk perception on health?; and what are the effective messages of the government’s campaign about disease prevention? As such, this study aimed to examine the perception of the public about the government’s campaign against COVID-19. Moreover, this study investigated the more effective messaging strategies for the campaign through subjective values, thoughts, and attitudes about the information dissemination, which became the basis for the degree of people’s participation in the disease prevention campaign.

**Method:**

In order to investigate the public perception on the campaign messages that are promoted by the government for prevention of COVID-19, this study implemented the Q methodology that studies subjective attributes of humans, unlike existing empirical studies. The Q methodology is an approach that endeavors to discover complex issues in human subjectivity through empirical studies. In order to determine the factors that trigger people’s voluntary and active practices and the motivation for disease prevention, the Q methodology is implemented to examine human subjectivity, thoughts, and attitudes. When it comes to the disease prevention campaigns that require strong civic awareness as members of the society, the rationale that induces people to participate in the campaign voluntarily and actively is based on their subjectivities, such as values, thoughts, and thinking. The voluntary awareness and behavior of the public campaign participants are based on their subjective perception about the given message.

**Results:**

In this study, it was ascertained that there were four different types of perceptions among Koreans on the message of the COVID-19 prevention campaign. The four perceptions are as follows: Type 1 is ‘the social threat caused by people with COVID-19 related symptoms;’ Type 2 is ‘the relational measures through personal hygiene;’ Type 3 is ‘the dependence on the social system due to the disease;’ and Type 4 is ‘the avoidance of the symptoms caused by human contact.’

**Conclusion:**

As a result of this study, it was possible to draw a correlation between people’s perception of the campaign message for COVID-19 prevention and campaign messages. The response method of the campaign message must be differentiated according to the type of people’s perception of the disease prevention campaign, and the message development required by stages. The different characteristics of each type are clearly explained by keywords: symptomatic person for Type 1, personal hygiene for Type 2, social system for Type 3, and etiquette for Type 4. Type 1 perceived the messages about symptomatic persons as important to prevent the disease spread in the community whereas Type 2 tried to protect themselves from physical threats by developing proactive prevention through personal hygiene management prior to infection. Type 3 responded actively by relying on social systems, such as medical institutions or management organizations, while Type 4 positively responded to the messages related to etiquette that allowed them to avoid virus infection caused by contact with others.

**Supplementary Information:**

The online version contains supplementary material available at 10.1186/s12889-022-13671-2.

## Introduction

On March 11, 2020, the World Health Organization (WHO) declared the novel Coronavirus (COVID-19) outbreak a global pandemic. COVID-19 was initially considered a novel coronavirus that would soon subside, similar to the Middle East Respiratory Syndrome-related coronavirus (MERS) and Zika virus disease. Contrary to expectations, COVID-19 spread rapidly around the world and although there are regional differences, pandemic situations have been a ongoing repetitive cycle with the emergence of mutations of the virus. Due to its rapid spread and high mortality rate, COVID-19 created a pandemic situation that has paralyzed social systems. The current pandemic situation, which has caused unprecedented social problems, is so stringent that it has dire situations that have led to the cessation of economic and human activities [11]. Fortunately, vaccinations against COVID-19 have begun in different countries from January 2021 (with various vaccine brands such as Pfizer, Moderna, AstraZeneca, Janssen, etc.). Which has allowed the situation to become much more stable; nevertheless, the number of confirmed cases is still on the rise and these circumstances seem to continue with the emergence of new mutant viruses. On July 7, 2021, Director-General Tedros Adhanom Ghebreyesus, of the WHO stated in a media briefing that “the world is at a perilous point in the current pandemic,” as COVID-19 deaths had passed the tragic milestone of four million recorded deaths (https://www.who.int/director-general/speeches/detail/director-general-s-opening-remarks-at-the-media-briefing-on-covid-19-7-july-2021). Recognizing the gravity of COVID-19, each country, including the G7 countries, have implemented quarantine measures but were inadequate in stopping the spread of the infection.

The Korean government acknowledged the severity of the disease on January 2020, when the domestic outbreak began. Korea has prepared and implemented various countermeasures which began on February 2020, including campaigns on preventive measures, rules on daily living, and basic guidelines on responses against COVID-19. On the beginning stages of the virus, due to the absence of accurate information about COVID-19, there were social confusions and mask shortages. To solve these problems, the government-led prevention campaign was launched to encourage the public to overcome the fear of COVID-19 and actively participate in preventative countermeasures. Through this campaign, it allowed for a social atmosphere that raised awareness in preventions for COVID-19 as the public voluntarily responded to it. In Korea, the efforts of government and private sectors to stop the spread of COVID-19 have continued by encouraging the public to take part in government campaigns, promoting rapid drive-through testing, and sharing information using IT. The dedicated efforts of medical staff and quarantine volunteers have also largely contributed to the fight against COVID-19.

Many questions have been raised in the ongoing battle against COVID-19: How does the public perceive the COVID-19 prevention campaign as a member of the society? What made the perception of the experts and the public on COVID-19 change from ‘simple’ to ‘serious’ epidemic? What is the risk perception on health? and what are the effective messages of the Korean government’s campaign about disease prevention? As such, this study aims to examine the perception of the public about the Korean government’s campaign against COVID-19. Moreover, this study investigated the more effective messaging strategies for the campaign through subjective values, thoughts, and attitudes about the information dissemination, which became the basis for Korean’s participation in the disease prevention campaign.

### The message of disease prevention campaign

Following the official declaration of COVID-19 as a pandemic by the WHO on March 11, 2020, international public health agencies have implemented stricter measures to mitigate or suppress the spread of the virus. Focusing on mitigation and containment policies carried out by major countries such as Canada, the United States, and some European countries have implemented various preventive campaigns from ‘virus containment strategies’ to ‘maintaining physical distance.’ Other examples include ‘strict personal hygiene,’ such as frequent hand washing, ‘physical and personal distancing,’ and ‘mask wearing’ in public places [2].

Doogan et al. [4] compares the guidelines of country-specific singularities about non-drug (non-vaccine) preventative campaigns. These comparisons were made between six countries (the United States, the United Kingdom, Canada, Australia, New Zealand, and Ireland) for the guidelines of: personal protection, social distancing, testing and tracking, blockade, workplace closure, and more. Particularly in the guidelines of ‘hand washing’ and ‘staying at home,’ the persuasive approach of the campaign was different for each individual country. The United States and Canada were much more receptive to hand hygiene than other countries, therefore, campaigns that encouraged people to wash hands while singing the ‘Happy Birthday’ song was a successful in the United States. Whereas in the U.K., the promotion of hand disinfection campaigns, with the use of alcohol based hand sanitizers, were much more successful. In the guidelines of the Australian government, it was stated that wearing a mask was not effective for the prevention of COVID-19 infections, but the people of Australia claimed otherwise. This shows the presence of conflict between the government’s campaign guidelines and the actual guidelines supported by the public. The guideline of ‘social distancing’ specifically ‘staying at home’ was taken very seriously in the U.S. and U.K. In the case of the U.K., this campaign was respected as a public duty rather than resistance.

Messages related to health and disease mainly use fear appeals that give shock and stimulation through threat or warning expression [17]. In addition to the degree and the magnitude of fear, such messages combine the concept of ‘self-harm’ and ‘other-harm’ [8, 12] to introduce a direction towards whether the subject of the threat appeal is ‘from oneself or from others’ and ‘is subject of harm or the one being harmed’. Also, public messages are classified according to the degree of social threat, which refers to the physical threats experienced by others, and ethical-moral threats that cause harm to others [5]. Schoenbachler and Whittler [15] states that the direction of message appeals, such as physical and social threats, are subdivided into psychophysiological, physio-psychosocial, and psychosocial threats which lead to more complex conceptualization.

The message frames of a disease are used to more clearly understand the public’s preferences on health-related messages. It is a pattern by the perception of the messages and about the power the public has in the process of health-related decision-making [10]. Various stories and news articles on disease prevention published by the government and public organizations provide the public with the appropriate information to respond to specific or infectious diseases. Although the related informative message serves its function as public promotion, there have been situations in which people have refused to take preventive actions [16]. This is because out of disbelief, people tend to acquire health-related information through unreliable sources through the internet, rather than getting the information from reliable sources such as medical professionals or medical journalists [3]. However, as the information about the disease is limited, incomplete, and sometimes inappropriate, the information provided are scarce, leading to unreliability. During the crisis of Acquired Immune Deficiency Syndrome (AIDS), the frame of ‘blockade’ was deployed to overcome the situation, focusing on the marginalized groups [19]. During the prevalence of Severe Acute Respiratory Syndrome (SARS), the focus was on the failed policies, using frames like ‘politics’ and ‘interest conflict’ on top of the pattern of overcoming the crisis [1]. The message conveyed by media tends to be contextualized by framing and then delivered as a newly conceptualized content. Therefore, it is paramount to focus on the frame formed by central organizations and the production of media messages need to centralize around these factual information to avoid confusion [11].

The messages for the campaign for disease prevention influence the response of perception through various health variables. In other words, a health variable also plays a major role in shaping the individual’s attitude and mindset [9]. For example, it can be a frame recognized by the public, as it can affect frame perception, and have a multifaceted tendency that can become the general structure of the frame.

## Method

The Q methodology is a tool to measure human subjectivity [13]. The effectiveness of public campaigns designed for disease prevention is not a unilateral message led by government policy; rather, it is related to the degree of people’s voluntary participation in the campaign message. The voluntary awareness and behavior of the campaign participants are based on their subjective perception about the given message. Behavior is a response to subjective values and perceptions. Subjectivity is the ‘subjective perspective on phenomena’ and includes all objects that exist in the inner world, such as the intrinsic disposition or taste of human as an observer [6]. In order to understand human beings, it is necessary to properly understand social phenomena or archetypal essence of men, while grasping the subjectivity essentially inherent in these elements. In order to investigate the public perception on the campaign messages that are promoted by the government for prevention of COVID-19, this study implemented the Q methodology that studies subjective attributes of humans, unlike existing empirical studies. When it comes to the campaigns for the prevention of diseases that require aggressive civic awareness for the members of society, the rationale that induces people to participate in the campaign voluntarily and actively is based on their subjectivities which include, values thoughts, and thinking. Therefore, this study examined the following issues: 1) How are the people’s perceptions on the COVID-19 prevention campaign message classified? 2) What are the differences between each type? and 3) What is the message strategy to increase the effectiveness of disease prevention campaigns? In addition, this study examined the association with previous studies that investigated the message strategy of the existing disease prevention campaigns.

### Q sample

A study of Q methodology begins with the development of a concourse. Various things such as statements, paintings, fragrances, etc. can be the subject of concourse, which also represents the world of communication about the topic [13]. A concourse refers to gathered statements about a topic, which is the general group of the items or shared opinions extracted from certain culture. It exists for every individual and may be shared with others, depending on the situation [6]. In order to compose the Q sample, the official campaign practice messages of the government were targeted from February 2020 to February 2021, which focus on the official posts on the homepages of: ‘Central Disaster Management Headquarters,’ ‘Central Disease Control Headquarters,’ and ‘Korea Centers for Disease Control and Prevention.’ There was a total of 1,479 campaign messages in the government guidelines during the time period, and the samples were collected focusing on the health campaigns to the public in accordance with the direction of research. Based on this, the final screening was conducted in consideration of some factors such as ‘preventive measures for individuals and households,’ ‘hygiene and immunity, face-to-face contact,’ ‘medical and disease-related issues,’ ‘guidelines for symptomatic persons, self-quarantine, and all citizens.’ As a result, a total of 33 representative statements were extracted.

### P sample

The P sample refers to the actual respondents with the subjective perspective. Because they are not simple research participants but a variable, the participants respond to the Q sample according to their defined point of view. Since the type of human subjectivity is not affected by the number of samples, it reflected Fisher’s structuring method for 32 P samples according to the small-sample doctrine of the Q methodology. For the P sample, 16 males and 16 females were selected in consideration of the gender ratio. Their composition by age included 16 people in their teens to 20 s, eight people in their 30 s to 40 s, and eight people in their 50 s to 60 s who searched the COVID-19 related news more than once up to five times a day.

### Ethical considerations

According to Article 13 (2) (http://irb.or.kr/menu02/commonDeliberation.aspx) of the Enforcement Rules of the Korea Institute for Bioethics Policy (KoNIBP), this study is excluded from ethical considerations because it does not specify the subject and does not collect or record sensitive information of individuals. In addition, all processes were conducted after sufficient verbal consent to each P sample for the purpose of research and the use of the results of the survey. In addition, research procedures, guarantees for anonymity and privacy were explained to each P sample.

### Q sorting and data processing

Q sorting is a process in which the respondent recognizes the relative importance of all Q samples by making the P sample distribute all Q samples within themselves. The relative importance of the respondents in the Q samples is accepted as an overall image of the research topic, and the subjectivity of the respondent intervenes in this process. In this study, the campaign messages that were selected as the Q samples were forcibly distributed by the respondents in the 9-point scale, based on the condition ‘What do you think is the most important response to prevent COVID-19?’ (See Table 1) During the Q sorting, an in-depth interview was conducted on the Q samples that were the most important (+ 4) and least important (-4) as shown in Table 1, and the interview details were utilized as basic data to interpret each type. After the Q sorting, the data was treated with QUANL statistics program after coding.

## Results

The results were derived through the ‘principal component analysis’ of the Q factor analysis. In addition, the correlation coefficients between factors were reviewed while factor weights were reflected. Also, explanatory variables and screen tests were additionally implemented to develop a comprehensive process for type classification. As a result, four types of eigenvalue of 1 or higher were finally discovered. The cumulative variables of the four types were found to be 59.40% with high explanatory power (see Table 2).

### Type 1: Social threats caused by symptomatic persons

Type 1 perceives as important the messages to be followed by people with COVID-19 symptoms, in order to prevent them from spreading the virus in the community. This type shows high involvement in the preventive measures related to others’ infection caused by symptomatic persons as reflected in these statements: ‘Those with symptoms should refrain from going out and avoid going to school or reporting for work (#25, z = 1.88)’; ‘If symptomatic, inform medical staff of all travel history overseas (if any) and any contact with persons who have had respiratory symptoms (#28, z = 1.83)’; ‘Those with symptoms should observe themselves for 3–4 days and get plenty rest at home (#26, z = 1.79)’; and ‘Those with symptoms should refrain from going out and visiting other areas in the country where COVID-19 is endemic (#29, z = 1.31)’. Above all, they prioritize blocking the number of cases that may harm others as it is a highly contagious disease. This is a characteristic similar to a social threat [5] that harms others ethically and morally. They feel the spread of the virus is a social threat, thus, they adhere to guidelines on social isolation and virus blockage, such as living in an isolated place (#31, z = 1.35), wearing masks when visiting medical institutions for treatment or other purposes (#20, z = 1.06), etc. (see Table 3).

*“You should stay at home if you have COVID-19 symptoms because it is contagious and may spread to everyone in the society. The only way to prevent the spread of infection from a symptomatic person is to avoid contact with him/her as much as possible. Because it is entirely based on individual autonomy, you must avoid contact with others if you have symptoms to prevent social spread (P1; P17; P22; P25).”*

These type of people are negative about the proactive preventive message for common diseases. As their interest is in the spread of the virus by symptomatic persons, the preventive messages related to proactive measures are not perceived as being important. They are not receptive to the messages related to individual practices for prevention, such as ‘Keeping a distance more than 2 m (at least 1 m) from others’ (#15, z = -1.46); ‘Disinfecting frequently touched items every day’(#7, z = -1.33); ‘Avoid visiting crowded places’(#12, z = -1.15), etc. The reasons that they are negative about ‘Refrain from eating and stay for only a short time when visiting places is inevitable’ (#13, z = -1.07) and ‘Restrict entry of outsiders as much as possible’ (#10, z = -1.02) is that such preventive measures cannot protect them from infection once symptoms appear. They are also negative about vaccination (#33, z = -1.08) (see Table 3), which is a powerful proactive measure against COVID-19, and thus agree with the non-drug prevention campaigns [4].

*“Even if we practice social distancing, isn’t it useless if an infected person is in a large crowd like public transportation or the office (classroom)? Since we are not sure whether we are infected with COVID-19, it is most important to block the infection from symptomatic persons (P15; P23).”*

The people in this type prefer the message guidelines that are directed to a specific target, such as symptomatic persons, rather than campaign messages targeting an unspecified majority like the entire nation. This is the stage of providing a customized program so that messages of the public campaign for disease prevention are provided directly to the targets. From the point of view of the messages, whether the subject of harm is from oneself or from others, these type of people tend to respond to other-harm messages. Furthermore, it is the type that gives importance to post-countermeasures messages.

### Type 2: Relational response through personal hygiene

Type 2 focuses on preventive messages designed to protect themselves in relation to the COVID-19 crisis. In order to prevent human-to-human transmission of the disease, they think it is more important to be careful about interpersonal actions causing direct contact rather than the government’s preventive measures. They think the following messages are important: ‘Wash hands with soap under running water for over 30 s or disinfect hands with sanitizer’ (#1, z = 2.45); ‘Cover your mouth and nose with your sleeve when coughing or sneezing’ (#2, z = 1.51); and ‘Avoid touching your eyes, nose, and mouth with unwashed hands’ (#3, z = 1.09). For these type of people, the following is recommend: immediately contact a call center or public health center for treatment if you have any symptoms of fever or unstable respiratory function (#30, z = 1.09); follow the instructions of the quarantine authorities if undergoing self-quarantine (#32, z = 1.33); and have a positive attitude toward proactive physical prevention to avoid further increase in confirmed cases (see Table 4). This type protects itself from the physical threats [5] posed by the disease by taking precautions through personal hygiene management even before becoming infected with COVID-19.

*“Hands are the dirtiest part of our body, so I think that frequent hand washing or disinfection can sufficiently prevent me from contracting the disease. In order to avoid suffering from physical pain caused by an infection, it is necessary to thoroughly manage personal hygiene. In order to maintain my health, I need to pay more attention to my hygiene first (P2; P4; P18; P27; P31).”*

Even though COVID-19 is a dangerous epidemic, this type believes that it is important to communicate carefully with others and maintain daily activities rather than blindly deterring exchanges with others. They negatively perceive the messages that ignore human sociality, although the COVID-19 pandemic is still ongoing. They are reluctant to communicate with others through phone or SNS to avoid directly meeting one another (#9, z = -2.00), and believe it is acceptable to work in a closed environment with others (#18, z = -1.68). Although they are sensitive to personal hygiene, they stress importance in sociality. For this reason, these type of people do not care much about borrowing others’ towels or other personal items such as cell phones (#8, z = -1.31) and do not care about disinfecting items after being used by others (#7, z = -1.22). They are also often unbothered about sharing food during mealtime or snacks (#14, z = -1.37) (see Table 4).

*“I think it is okay to meet people as long as you follow the quarantine rules. Social distancing for more than two meters and wearing a mask will prevent the spread of infection. You can’t stop interacting with people and socializing because of the fear of infection (P3; P8; P32).”*

These are the type of people that want to maintain social relationships through human exchanges by implementing preventive actions that become the guidelines for personal hygiene management in their daily lives. These people consider the universality of behavioral aspects to improve the health status of the targets in a public campaign. To the public that is not much sensitive to the disease, a message that can be easily understood and actively practiced in the current situation is more effective than the one that creates a high level of fear. When it comes to judging the victims, these people respond to the message appeal of ‘self-harm’ that weighs on “Am I the harming object?”. In terms of proactive measures and post-countermeasures, it is a type that gives importance to proactive messages.

### Type 3: Dependence on social systems due to the disease

Type 3 relies on social systems, such as medical institutions or management authorities in relation to the transmission of COVID-19, with a focus on the messages of active response against the infection. The emphasis is on the containment and mitigation of the virus by the health environment and institutional systems, rather than implementing personal hygiene or social distancing. Those with suspected or confirmed symptoms of COVID-19 must rely on the institutional environments or medical institutions to prevent transmission and provide treatment. These type of people is very receptive to messages like ‘Those subject to self-quarantine must strictly follow the instructions of medical personnel and quarantine authorities (#32, z = 1.77)’; ‘Those with symptoms should refrain from going out and visiting other areas in the country where COVID-19 is endemic (#29, z = 1.39)’; and ‘If you develop fever and respiratory symptoms, contact a call center or public health center and visit a screening clinic (#30, z = 1.52)’. To prevent the spread of COVID-19, they are willing to stay alone in isolated places (#31, z = 1.74), avoid going out (#25, z = 1.00), and prioritize getting a COVID-19 vaccine, rather than other available vaccinations such as pneumococcal and influenza vaccines (#33, z = 1.92). Regarding the persons with symptoms, they perceive as essential the messages like ‘Those with symptoms should use their own car when visiting medical institutions’ (#27, z = 1.27) or ‘If symptomatic, inform medical staffs of overseas travel history (if any) and possible contact with persons who have had respiratory symptoms’ (#28, z = 1.22) (see Table 5). Unlike Type 1, Type 3 attaches great importance to vaccination and believes that disease should be managed and controlled professionally and systematically.

*“I think vaccination is the best way to prevent coronavirus infection. Many healthcare professionals say that vaccination can not only stop the spread of infection to society, but also lower the risk of having a serious condition after contracting the disease. In particular, those with high-risk conditions should be controlled and managed by following the instructions of the quarantine authorities or medical institutions rather than following their own judgment. An effective social management system is needed to cope with the coronavirus (P12; P13; P14; P16; P19; P20).”*

They are more interested in the actions to be taken after being infected with COVID-19 rather than proactive prevention. The messages on universal simple disease preventive measures are not perceived as important. They dismiss medication or checking medical treatment schedules of those with chronic disease as separate matters (#23, z = -1.58), and a balanced diet, regular exercise, and sufficient sleep are not important at this point (#4, z = -1.55). These type of people are willing to accept the professional and systematic messages on the coronavirus symptoms (see Table 5).

*“I am not interested in any contents that are not related to the guidelines of medical institutions or government authorities to respond to the coronavirus. I think the only way to quickly overcome this situation is following the preventive rules against the virus infection guided by the government or medical institutions (P13).”*

Type 3 takes the messages of systematic countermeasures from the government or medical institutions as being important due to the increased number of COVID-19 confirmed cases and the high fatality rate. They value the practical quarantine measures focused on COVID-19 and the messages guiding the persons confirmed to be infected with COVID-19. The public campaigns related to this should establish a customized program so that a direct message can be delivered to the targets, which are conscious of the confirmed persons. From the perspective of the message regarding the subject of harm, a message appealing to ‘other-harm’ is more effective in this type. They are more sensitive to the messages of post-countermeasures than proactive ones and tend to trust and follow professional and systematic social management systems.

### Type 4: Avoidance of the symptoms caused by personal contact

Type 4 gives importance to preventive messages related to viral infection through contact with others. In particular, these type of people respond sensitively to airborne transmission by droplet and respiratory symptoms, while emphasizing personal etiquette, wearing a mask, and physical distancing to avoid the symptoms caused by the virus. They think that it is good to refrain from talking loudly and singing when other people are around (#17, z = 1.93) and it is considered basic etiquette to cover one’s mouth with their sleeves when coughing or sneezing (#2, z = 1.75). Moreover, they try to avoid contact with persons who have fevers or respiratory symptoms (#16, z = 1.89), always wear a mask when going to medical institutions (#20, z = 1.37), and will follow the instructions of medical personnel and quarantine authorities (#32, z = 1.30) (see Table 6).

*“I am very careful when coughing or sneezing or when I am in a crowded place, as you can easily get infected with coronavirus. I heard that the coronavirus may be spread through airborne transmission, so I am always careful where there are other people around (P6; P10; P26).”*

They respond positively to the messages about social distancing and minimizing exposure to the virus, but they hope to secure their personal information and privacy. They respond negatively to the use of QR codes or electronic access systems when visiting public places (#19, z = -1.32) because they believe that these measures, by the government, to prevent the spread of the virus are a violation to individual human rights. Although prevention is the best option, they believe it is unnecessary to live isolated from the outside world, even if COVID-19 is spreading (#10, z = 1.39), as beliefs of personal life and freedom should be maintained with the normal scope of daily life (see Table 6).

*“After the outbreak of COVID-19, many guidelines related to its prevention have been introduced. I think it is important to follow all the guidelines to avoid getting infected with the virus, but sometimes it's difficult to follow them all. Wouldn’t it be better if I take care of myself according to my lifestyle and situation? (P10; P24)”*

They are a type that corresponds to the stage of considering the universality of the behavioral aspect to improve the health status of the targets in a public campaign. These people prefer the macroscopic messages guiding the public that are relatively less sensitive to epidemics by inculcating the idea that all areas apply to oneself. These types of people perceive the virus from its symptoms and determine their attitudes and behaviors, regardless of whether the symptoms are from others or from themselves. This type is more sensitive to the messages with specific symptoms and countermeasures of the disease rather than figuring out the subjects of the infection. They correspond to the initial stage of post-countermeasures rather than proactive prevention.

## Discussion and implications

The directions suggested by the various messages on the disease prevention campaigns do not always draw positive perceptions and effective responses from the public. What is perceived as “positive” or “negative” varies from person to person. The frames created by themselves may be more effective than the ones given by others, such as the media or government. Therefore, ‘what people chose’ can result in more positive efficiency than ‘what is chosen.’ The health goals chosen by the people reinforce such perceptions and can be practiced in their daily lives as a habit [7]. The success of public campaigns on disease prevention depends on how the state or related organizations reflect their messages into the subjective perceptions of the people. In connection with the COVID-19 prevention campaigns, personal hygiene, physical distancing, and vaccination were used as the message frames, along with various situational contexts. The shift in the people’s risk perception regarding COVID-19 from simple to serious epidemic does not imply the implementation of what is stipulated in the frame. It rather indicates that people’s perception on existing or newly created frames have changed according to the situations caused by the spread of COVID-19. Besides, the case of Korea can be referred to by governments and communication officials from other countries to take a new perspective on COVID-19 response strategies.

As a result of this study, it was possible to draw a correlation between people’s perception of the campaign message for COVID-19 prevention and campaign messages (see Table 7). Therefore, the response method of the campaign message must be differentiated according to the type of people’s perception of the disease prevention campaign, and the message development required by stages. The different characteristics of each type are clearly explained by keywords: symptomatic person for Type 1, personal hygiene for Type 2, social system for Type 3, and etiquette for Type 4. Type 1 perceived the messages about symptomatic persons as important to prevent the disease spread in the community whereas Type 2 tried to protect themselves from physical threats by developing proactive prevention through personal hygiene management prior to infection. Type 3 responded actively by relying on social systems, such as medical institutions or management organizations, while Type 4 positively responded to the messages related to etiquette that allowed them to avoid virus infection caused by contact with others.

With respect to the subjects of ‘harm’ studied by Peak and Hove [12], it was possible to identify the positive correlation between Type 1 and Type 3 with ‘other-harm’ and Type 2 with ‘self-harm.’ Type 2, which emphasizes personal hygiene, prioritizes proactive prevention and is exposed to the first stage of the disease prevention campaigns. The universal messages on practice related to personal hygiene are required at this stage. Type 4 is sensitive to the infection through contact and accepts messages on post-countermeasures in the second stage of the campaign, when the disease spread in the society has progressed to some extent. When it comes to contact caused by personal interchanges, the messages related to the observations of universal behaviors are required. Types 1 and 3 tend to be exposed to the messages about post-countermeasures in the third stage of the campaign, in which the social spread of disease has become serious. These types require customized messages constructed for each individual situation or singular target for disease treatment. However, there is a difference between Type 1 and Type 3, as Type 1 focuses on symptomatic individuals whereas Type 3 focuses on messages related to the social system.

The message of a public campaign should be flexible and multifaceted rather than uniform to achieve a successful campaign. In a previous study, the messages shared at a certain point of time naturally constituted the social frame and then used for message framing as a belief or symbol [14]. Various preventive campaigns, from virus containment strategies to physical distancing and vaccination, were used as message frames in accordance with the situation [2]. Nonetheless, the message frame of disease prevention campaigns did not always work positively. Sometimes, negative message frames were made with limited, incomplete, and inappropriate contents. During the AIDS crisis, a blockade frame was used [19] while using the frames of politics and interest conflict with the pattern of overcoming a crisis during the SARS outbreak [1]. Some frames emphasized specific themes or episodes [18]. In the public campaigns for disease prevention, it is difficult to generalize the message framing in a light and simple way because the success of the campaigns cannot be guaranteed without considering individual subjectivity and diversity. According to the result of the study, the types of people’s perceptions on the messages of prevention campaigns amidst the COVID-19 pandemic differ by characteristic. The difference in the aspects that people perceive as important for the same disease is clear. The purpose of the public campaign for disease prevention is to overcome disease through the spread of social participation, rather than production of issues through message framing. Such campaigns should be approached in terms of responding to the messages by target and stage. In addition, it should be taken into account that subjective perceptions on disease and its prevention among people differ depending on political, social, and cultural values of each society.

**Table 1:** Q-sort distribution (*N*=33)

most unimportant					most important			
-4	-3	-2	-1	0	+1	+2	+3	+4

**Table 2:** Eigenvalue, Variance, Cumulative percentage and Correlations between Types

	Type1 (*N* = 6)	Type2 (*N* = 13)	Type3 (*N* = 8)	Type4 (*N* = 5)
Eigenvalue	11.6193	3.1078	2.5339	1.7460
Variance (%)	0.3631	0.0971	0.0792	0.0546
Cumulative percentage (%)	0.3631	0.4602	0.5394	0.5940
Type1	1.0000	0.612	0.503	0.487
Type2	0.612	1.0000	0.443	0.544
Type3	0.503	0.443	1.0000	0.422
Type4	0.487	0.544	0.422	1.0000

**Table 3:** Q Statement with Z-score of ± 1.00 or higher in Type 1

Q statement	Z-score
25. Those with COVID-19 symptoms should refrain from going out and avoid going to school or reporting for work	1.88
28. If symptomatic, inform medical staff of all travel history overseas (if any) and any contact with persons who have had respiratory symptoms	1.83
26. Those with COVID-19 symptoms should be under observation for 3–4 days and get plenty rest at home	1.79
31. Those undergoing self-quarantine should live in an isolated place	1.35
29. Those with COVID-19 symptoms should refrain from going out and visiting other areas in the country where COVID-19 is endemic	1.31
20. Wear a mask when visiting medical institutions	1.06
6. Check body temperature with a thermometer	-1.01
10. Restrict entry of outsiders as much as possible	-1.02
18. Focus on individual play rather than group play	-1.04
13. When visiting a place is inevitable, refrain from eating and stay for only a short period	-1.07
33. Get vaccinated against COVID-19	-1.08
12. Avoid visiting crowded places	-1.15
7. Disinfect frequently touched items every day	-1.33
15. Keep a distance more than 2 m (at least 1 m) from others	-1.46
9. Communicate with friends by phone or SNS	-1.82

**Table 4:** Q Statement with Z-score of ± 1.00 or higher in Type 2

Q statement	Z-score
1. Wash hands with soap under running water for over 30 s or disinfect hands with sanitizer	2.45
2. Cover your mouth and nose with your sleeve when coughing or sneezing	1.51
20. Wear a mask when visiting medical institutions	1.44
32. Those undergoing self-quarantine must strictly follow the instructions of medical personnel and quarantine authorities	1.33
30. If you develop fever and respiratory symptoms, contact a call center or public health center and visit a screening clinic	1.09
3. Avoid touching your eyes, nose, and mouth with unwashed hands	1.09
10. Restrict entry of outsiders as much as possible	-1.01
7. Disinfect frequently touched items every day	-1.22
8. Do not share personal items (towels, tableware, mobile phones, etc.)	-1.31
14. Don't share food	-1.37
18. Focus on individual play rather than group play	-1.68
9. Communicate with friends by phone or SNS	-2.00

**Table 5:** Q Statement with Z-score of ± 1.00 or higher in Type 3

Q statement	Z-score
33. Get vaccinated against COVID-19	1.92
32. Those undergoing self-quarantine must strictly follow the instructions of medical personnel and quarantine authorities	1.77
31. Those undergoing self-quarantine should live in an isolated place	1.74
30. If you develop fever and respiratory symptoms, contact a call center or public health center and visit a screening clinic	1.52
29. Those with COVID-19 symptoms should refrain from going out and visiting other areas in the country where COVID-19 is endemic	1.39
27. Those with COVID-19 symptoms should use their own car when visiting medical institutions	1.27
28. If symptomatic, inform medical staff of all travel history overseas (if any) and any contact with persons who have had respiratory symptoms	1.22
25. Those with COVID-19 symptoms should refrain from going out and avoid going to school or reporting for work	1.00
18. Focus on individual play rather than group play	-1.03
9. Communicate with friends by phone or SNS	-1.19
21. Make sure you have necessary vaccinations such as pneumococcal and influenza vaccines	-1.51
4. Eat balanced meals, exercise regularly, and get enough sleep	-1.55
23. If you have a chronic disease, take your medicine at a set time and keep a medical schedule	-1.58

**Table 6:** Q Statement with Z-score of ± 1.00 or higher in Type 4

Q statement	Z-score
17. Avoid loud conversations, singing, and other activities which are prone to producing respiratory aerosols (droplets) that cause infection	1.93
16. Avoid contact with people who have fever or respiratory symptoms	1.89
2. Cover your mouth and nose with your sleeve when coughing or sneezing	1.75
20. Wear a mask when visiting medical institutions	1.37
32. Those undergoing self-quarantine must strictly follow the instructions of medical personnel and quarantine authorities	1.30
6. Check body temperature with a thermometer	-1.20
19. Use QR codes and electronic access system when visiting public places	-1.32
10. Restrict entry of outsiders as much as possible	-1.39
14. Don’t share food	-1.44
7. Disinfect frequently touched items every day	-1.62
4. Eat balanced meals, exercise regularly, and get enough sleep	-1.71

**Table 7:** Correlation between the characteristics of each type and campaign messages

Type	Keyword	Subject of ‘harm’^a^	Proactive prevention vs. Post-countermeasures	Stage of campaign	Message type
Type 1	Symptomatic individuals	Other-harm	Post-countermeasures	Stage 3	Target-customized
Type 2	Personal hygiene	Self-harm	Proactive prevention	Stage 1	Universal behaviour
Type 3	Social system	Other-harm	Post-countermeasures	Stage 3	Target-customized
Type 4	Etiquette	Not related	Post-countermeasures	Stage 2	Universal behaviour

^a^[12]

## Supplementary Information

**Additional file 1.** Q Sample 

## Data Availability

Not applicable. The datasets used and/or analyzed during the current study are available from the corresponding author on reasonable request.
