# Supplementary material for: Strategic exploration of the COVID-19 prevention campaign message: based on South Koreans’ perception type
Source: BMC Public Health. 2022 Jun 28;22:1262. doi: 10.1186/s12889-022-13671-2 (PMC9238254; doi:10.1186/s12889-022-13671-2)
Supplement: Supplementary file 1 — Additional file 1. Q Sample [file 12889_2022_13671_MOESM1_ESM.docx]

# Appendix 1 - Q Sample

1. Wash hands with soap under running water for over 30 seconds or disinfect hands with sanitizer
2. Cover your mouth and nose with your sleeve when coughing or sneezing
3. Avoid touching your eyes, nose, and mouth with unwashed hands
4. Eat balanced meals, exercise regularly, and get enough sleep
5. Ventilate regularly
6. Check body temperature with a thermometer
7. Disinfect frequently touched items every day
8. Do not share personal items (towels, tableware, mobile phones, etc.)
9. Communicate with friends by phone or SNS
10. Restrict entry of outsiders as much as possible
11. Avoid unnecessary outings, gatherings, and travel
12. Avoid visiting crowded places
13. When visiting a place is inevitable, refrain from eating and stay for only a short period
14. Don't share food
15. Keep a distance more than 2m (at least 1m) from others
16. Avoid contact with people who have fever or respiratory symptoms
17. Avoid loud conversations, singing, and other activities which are prone to producing respiratory aerosols (droplets) that cause infection
18. Focus on individual play rather than group play
19. Use QR codes and electronic access system when visiting public places
20. Wear a mask when visiting medical institutions
21. Make sure you have necessary vaccinations such as pneumococcal and influenza vaccines
22. If breathing is difficult, take off the mask in a space where distancing is possible and then take a rest
23. If you have a chronic disease, take your medicine at a set time and keep a medical schedule
24. Those with high-risk conditions should stay at home if possible
25. Those with COVID-19 symptoms should refrain from going out and avoid going to school or reporting for work
26. Those with COVID-19 symptoms should be under observation for 3-4 days and get plenty rest at home
27. Those with COVID-19 symptoms should use their own car when visiting medical institutions
28. If symptomatic, inform medical staff of all travel history overseas (if any) and any contact with persons who have had respiratory symptoms
29. Those with COVID-19 symptoms should refrain from going out and visiting other areas in the country where COVID-19 is endemic
30. If you develop fever and respiratory symptoms, contact a call center or public health center and visit a screening clinic
31. Those undergoing self-quarantine should live in an isolated place
32. Those undergoing self-quarantine must strictly follow the instructions of medical personnel and quarantine authorities
33. Get vaccinated against COVID-19
